# Analysis of the influence of antithrombin on microvascular thrombosis: anti-inflammation is crucial for anticoagulation

**DOI:** 10.1186/s40635-015-0058-x

**Published:** 2015-07-09

**Authors:** Heiko Sorg, Julius O. Hoffmann, Johannes N. Hoffmann, Brigitte Vollmar

**Affiliations:** Institute for Experimental Surgery, University Medicine Rostock, Schillingallee 69a, 18057, Rostock, Germany; Department of Plastic, Reconstructive and Aesthetic Surgery, Handsurgery, Alfried Krupp Krankenhaus, 45276 Essen, Germany; Division of Vascular Surgery, Universitätsklinikum Essen and Contilia Heart and Vascular Center, 45147 Essen, Germany

**Keywords:** Thrombosis, Microvasculature, Light-dye model, Ferric chloride, Cremaster muscle

## Abstract

**Purpose:**

Microvascular thrombosis during septic conditions is of essential clinical relevance, but the pathomechanisms are not yet completely understood. The purpose of this study was to study the distinguished differentiation of the interactions of inflammation and coagulation using antithrombin (AT), a mediator of anticoagulation and anti-inflammation.

**Methods:**

Using a thrombosis model in a cremaster muscle preparation of male C57Bl/6J mice (*n* = 83), we quantitatively assessed microvascular thrombus formation by using intravital fluorescence microscopy. Experimental groups consisted of animals treated with AT or with tryptophan^49^-blocked AT (TrypAT), which exerts only anticoagulant but no anti-inflammatory effects. To further see whether endothelial glycosaminoglycan (GAG) binding with consecutive prostacyclin (PGI_2_) release is mandatory for the anticoagulant process of AT, animals were administered heparin or indomethacin either alone or in combination with AT.

**Results:**

The antithrombotic capacity of AT significantly differs in the experimental groups in which anti-inflammation was antagonized. This is given by the significantly prolonged occlusion times (*p* < 0.05) and higher patency rates in case of application of AT alone; while all other groups in which the anti-inflammatory action of AT was blocked by TrypAT, heparin or indomethacin revealed thrombus kinetics comparable to controls.

**Conclusions:**

The anti-inflammatory influence of AT is essentially linked to its anticoagulant effect in the microvascular system. Those specifications of the active profile of AT characterize the intimate interactions of the anticoagulant and anti-inflammatory pathways. This might be of relevance for AT as a therapeutic agent in critically diseased patients and the clinical understanding of microvascular thrombosis.

## Background

The close interactions of coagulation and inflammation in the microvascular system are not yet understood in total but are of high relevance especially for the treatment of sepsis, septic shock, and associated phenomena like disseminated intravascular coagulation (DIC). During this complex and fast-acting pathomechanism in septic disorders, the levels of coagulation markers like antithrombin (AT) or activated protein C (APC) change significantly and can therefore be used as powerful prognostic markers of DIC [1, 2]. In this context, the expectations on the high-dosed supplementation of antithrombin (AT), one of the most important human physiologic coagulation inhibitors, were once high. AT application, however, could not induce a significant survival benefit in the KyberSept-trial [3] and is, therefore, globally not recommended in sepsis therapy guidelines [4, 5]. The failure of AT might have been linked to its specific interaction to heparin, as it could be shown that heparin irreversibly blocks the binding site of AT at the endothelium, inhibiting the anti-inflammatory release of prostacyclin [6, 7]. Furthermore, this close interaction could also be demonstrated in a prospectively defined subset of patients with high-dosed AT in the KyberSept-trial, who did not receive concomitant heparin-treatment for thrombosis prophylaxis [3, 6–8]. Only in the AT alone group mortality has been significantly reduced, suggesting an adverse reaction of AT with heparin [3, 6–8]. Recently, it could be shown that moderate doses of AT in septic patients could increase the patient recovery from DIC without major bleeding disorders [9]. In parallel, our group was also able to show in experimental models that high-dose AT application without heparin sufficiently prevented thrombosis under physiological as well as endotoxemic conditions [10, 11]. AT is a well-explored mediator with a dual profile of action including its anti-inflammatory as well as anticoagulant effects. The aim of the following study, therefore, was to further elucidate interactions between the coagulation and inflammatory system in the microvasculature using AT as lead substance due to its influence on both systems and to further evaluate which of AT’s active profiles are crucial for the anticoagulant effectiveness.

## Methods

### Animals

A total of 83 male C57Bl/6J mice (10–12 weeks old, 20–30 g body weight (bw)) have been used for the study. The animals were housed in standard laboratories with a 12 h light-dark cycle and had ad libitum access to chow and water. All experiments were conducted in accordance with guidelines for the Care and Use of Laboratory Animals and the Institutional Animal Care and Use Committee (University of Rostock, Medical Faculty, Rostock, Germany).

### Mouse cremaster muscle preparation

For the study of microvascular thrombus formation in vivo, we used the open cremaster muscle preparation, as previously described by our group [10, 11]. Mice were anesthetized by an intraperitoneal injection of ketamine (90 mg/kg bw) and xylazine (25 mg/kg bw). Prior to the preparation of the cremaster muscle, animals were placed on a heating pad and the insertion of a fine polyethylene catheter (PE10, 0.28 mm internal diameter) into the right jugular vein served to apply drugs and fluorescent dyes. A midline incision of the skin and fascia was made over the ventral aspect of the scrotum and extended up to the inguinal fold and to the distal end of the scrotum. The incised tissues were retracted to expose the cremaster muscle that was maintained under gentle traction to carefully separate the remaining connective tissue by blunt dissection from the cremaster. Afterwards, the cremaster muscle was incised, avoiding cutting of larger anastomosing vessels. Hemostasis was achieved with 5–0 threads, which also served to spread the muscle. After dissection of the vessel connecting the cremaster and the testis, the epididymis and testis were put to the side of the preparation. After preparation of the cremaster muscle, the animals were allowed to recover from surgery for 15 min.

### Intravital microscopy and thrombus induction

After IV injection of 0.1 mL 5 % fluorescein isothiocyanate (FITC)-labeled dextran (MW 150.000 Da, Sigma), 0.1 mL 0.2 % rhodamine 6G (MW 476 Da, Sigma), and subsequent circulation for 30 s, the cremaster muscle microcirculation was visualized by intravital fluorescence microscopy using a Zeiss microscope (Zeiss Axiotech Vario, Jena, Germany). The epi-illumination setup included a 100 W HBO mercury lamp and an illuminator equipped with a blue (450 to 490 nm/>520 nm excitation/emission wavelengths) and green filter set (530 to 560 nm/>580 nm). Microscopic images were recorded by a charge-coupled-device video camera (FK 6990A-IQ, Pieper, Berlin, Germany) and stored on videotapes for off-line evaluation. Using a ×63 water immersion objective (Achroplan ×20/0.5 W, Zeiss), blood flow was monitored in individual venules. Thrombus formation was induced in randomly chosen venules by constant superfusion with 25 μL ferric chloride every 60 s (12.5 mmol/L; Sigma-Chemical, Deisenhofen, Germany). Additionally, the light-dye model was used in which the exposure of FITC-dextran to blue light results in the release of free oxygen radicals and consecutive damage of the endothelial cell. Recording of vessels was stopped after blood flow in the vessel ceased for at least 45 s due to complete vessel occlusion (CVO).

### Microcirculatory analysis

Kinetics of intravascular thrombus formation has been quantified off-line by analyzing the videotaped images using the computer-assisted image analysis system CapImage (Dr. Zeintl Software, Heidelberg, Germany). Thrombus formation was quantified by assessing the time until sustained cessation of blood flow due to complete vessel occlusion, as well as the percentage of vessels which were completely clogged after 20 min of continuous thrombus induction. If a vessel did not clog within 20 min of continuous ferric chloride exposure, the observation was stopped, and the vessel was considered as patent. Venular patency rate describes the patent vessels in percent of all venules under investigation. Microcirculatory analysis further included the determination of vessel diameter, thrombus height, red blood cell velocity (RBCV), and additionally, the calculation of vascular wall shear rates (*γ*), based on the Newtonian definition *γ* = 8 × *V*/*D*, with *V* representing the red blood cell centerline velocity divided by 1.6, according to the Baker-Wayland factor, and *D* representing the individual inner vessel diameter.

### Experimental design and experimental groups

**Fig. 1:**
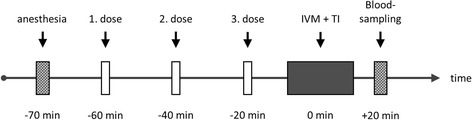
In vivo protocol experimental groups. Flow chart displaying the in vivo protocol for the different experimental groups in C57B1/6J mice. Before intravital fluorescence microscopy (IVM) and thrombosis induction (TI), anesthetized mice received different applications of the respective substances as mentioned in the dosing regimen of Table 1

Twenty minutes before induction of thrombosis (TI), animals received either a single IV-bolus of AT (250 IU/kg bw; Kybernin HS, CSL Behring, Marburg, Germany; *n* = 16), or saline (control; *n* = 16) corresponding to the fluid amount applied in the verum experiments (Fig. 1, Table 1). In a third group of animals, tryptophan^49^-blocked AT (TrypAT; 250 IU/kg; CSL Behring, Marburg, Germany; *n* = 9) was administered 20 min before TI (Fig. 1). Chemical modification of a single tryptophan residue in AT was performed according to the method of Blackburn et al. [12]; Tryp^49^ was labeled by using dimethyl (2-hydroxy-5-nitrobenzyl) sulfonium bromide. Because Tryp^49^-blocked AT evokes equivalent anticoagulant effects, considering actions depending on the progressive inhibition of proteases and simultaneously shows inability to interact with glycosaminoglycans (GAG), this tool allowed us to discriminate between AT effects on the coagulation and on the microcirculation, i.e., anti-inflammatory ability of non-blocked AT. To elucidate the distinct anti-inflammatory effects of AT on venous thrombus formation and the interaction with prostacyclin, we examined the cyclooxygenase inhibitor indomethacin (5 mg/kg sc; Liometacen, Promedica, Parma, Italy) in combination with AT (Indo + AT, *n* = 16). In addition, heparin (100 IU/kg bw; Liquemin N, Hoffmann-La Roche AG, Grenzach-Wyhlen, Germany) was combined with AT (Hep + AT; *n* = 8). The combination of AT and heparin was applied to study whether the antithrombotic effect of AT is essentially linked to the endothelial GAG binding. As respective control groups, single heparin (*n* = 9) and indomethacin treatment (*n* = 9) has been studied in the dosages as mentioned above (Fig. 1).

**Table 1:** In vivo protocol and experimental groups

Groups	1. dose	2. dose	3. dose
	Subcutaneous	Intravenous	Intravenous
Control	NaCl (0.9 %)	NaCl (0.9 %)	NaCl (0.9 %)
AT	NaCl (0.9 %)	NaCl (0.9 %)	AT (250 IU/kg)
TrypAT	NaCl (0.9 %)	NaCl (0.9 %)	TrypAT (250 IU/kg)
Indo	indo (5 mg/kg)	NaCl (0.9 %)	NaCl (0.9 %)
Indo + AT	indo (5 mg/kg)	NaCl (0.9 %)	AT (250 IU/kg)
Heparin	NaCl (0.9 %)	heparin (100 IU/kg)	NaCl (0.9 %)
Heparin + AT	NaCl (0.9 %)	heparin (100 IU/kg)	AT (250 IU/kg)

Flow chart displaying the in vivo protocol for the different experimental groups in C57Bl/6J mice. Before intravital fluorescence microscopy (IVM) and thrombosis induction (TI), anesthetized mice received different applications of the respective substances

*AT* antithrombin, *Indo* indomethacin, *TrypAT* tryptophane^49^-modified AT

### Statistical analysis

Data were analyzed for normality and equal variance across the groups. Differences between groups were assessed using 1-way ANOVA followed by the appropriate post-hoc comparison test (all pairwise). All data revealed normal distribution and were expressed as means ± SEM. Overall statistical significance was set at *p* < 0.05. Statistics and graphics were performed using the software packages SigmaStat and SigmaPlot (Jandel Corporation, San Rafael, CA, USA).

## Results

### Thrombus formation

**Fig. 2:**
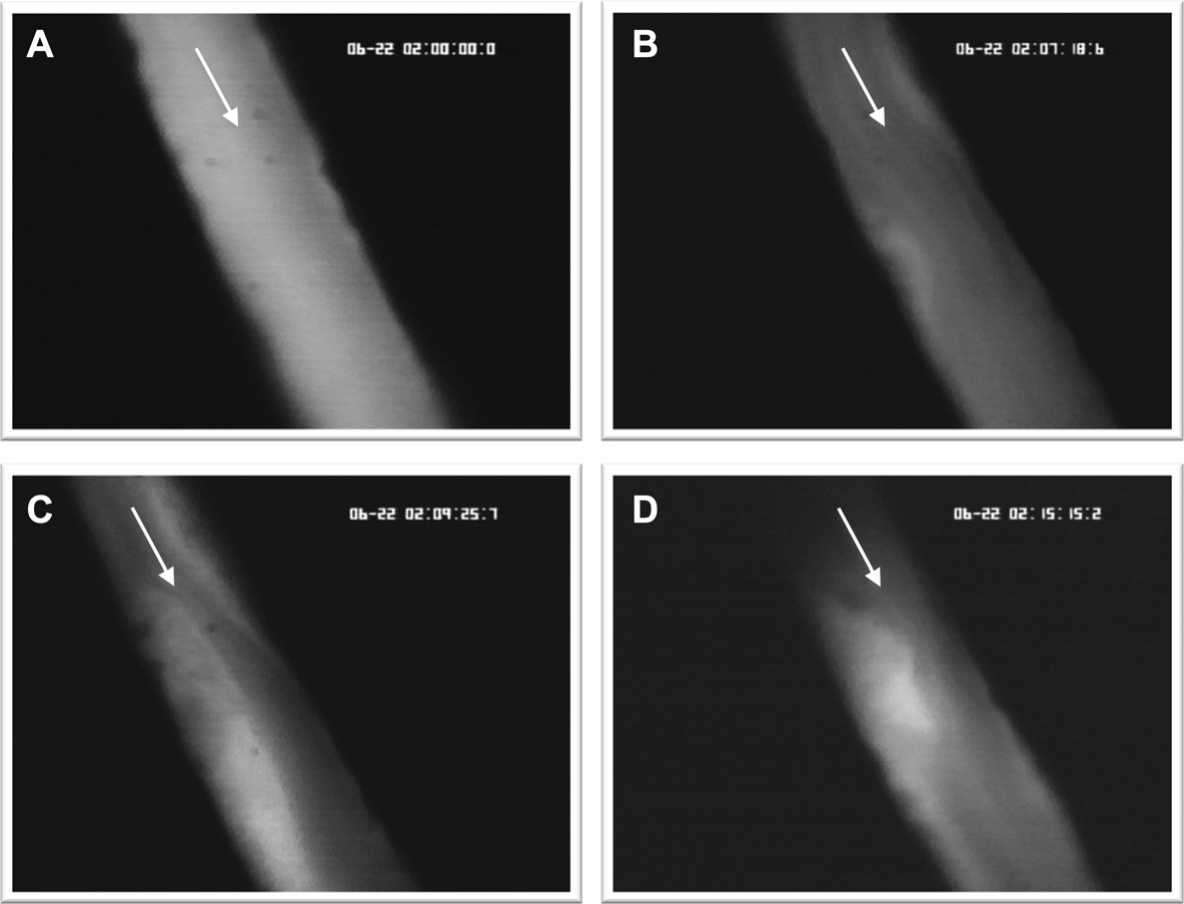
Thrombus growth. Representative intravital microscopic images of a mouse cremaster venule showing a growing thrombus at four different time points (*A* = 0 s; *B* = 438 s; *C* = 565 s; *D* = 915 s) during thrombus induction (magnification ×630). The arrows indicate the direction of the blood flow

With continuous ferric chloride superfusion and light exposure, thrombus formation was initiated, and a constant thrombus growth has been observed (Fig. 2). To analyze the influence on microcirculatory parameters, we acquired values for thrombus height, *RBCV*, and wall shear rate. The relation between RBCV and thrombus height demonstrates a negative correlation throughout all groups. With increasing thrombus height, the RBCV is decreasing (data not shown; y = −0.02 × +1.3; *r* = 0.65). Within these characteristics, the individual groups did not significantly differ to each other showing standardized TI among all groups. The RBCV is constantly decreasing in all groups from ~1.0 mm/s to ~0.65 mm/s during the time of continuous TI. Furthermore, there was no significant influence on the results of the wall shear rate between all groups.

### Patency rate

**Fig. 3:**
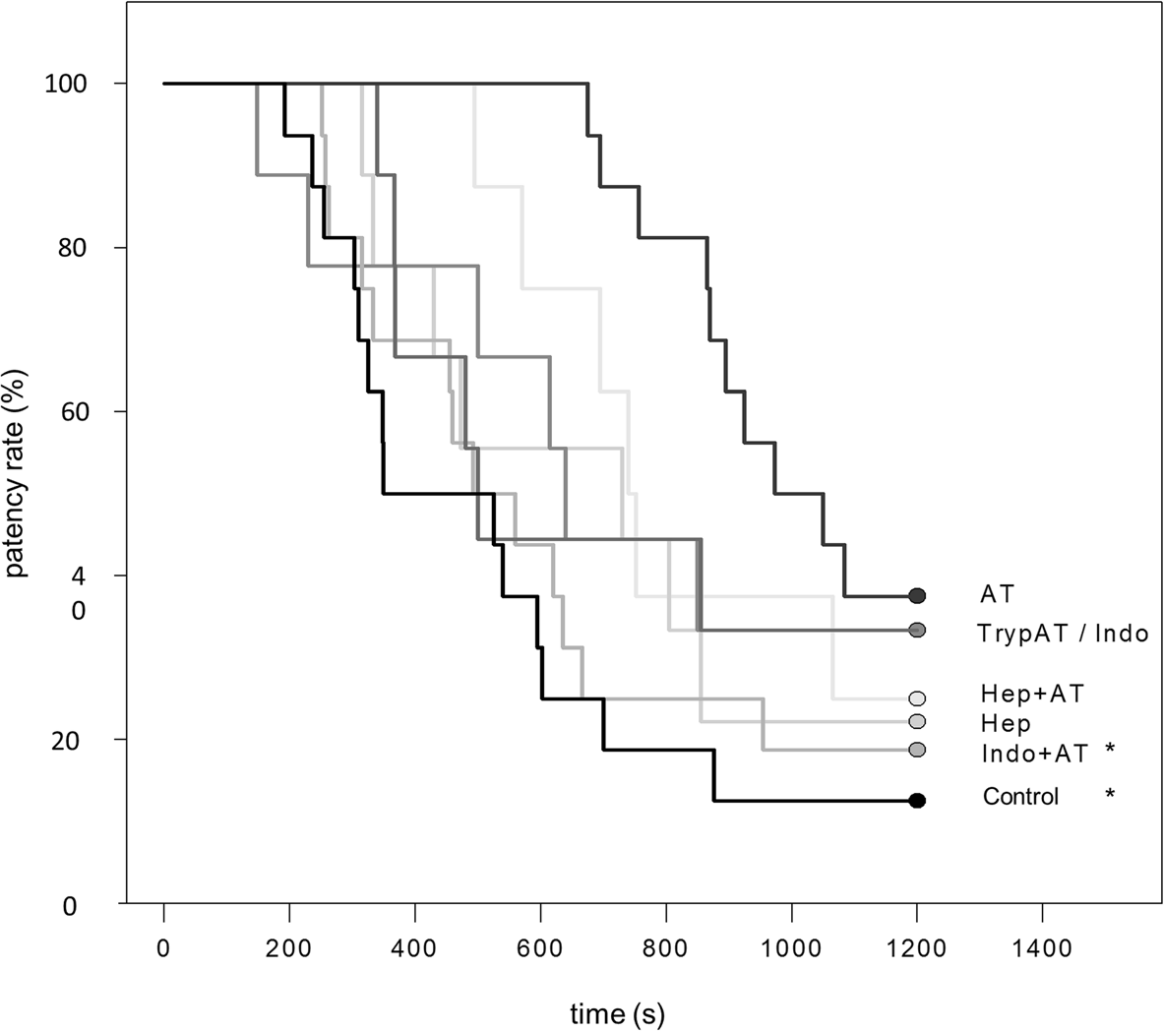
Patency rate of venules. Cumulative Kaplan-Meier patency rate of venules after induction of thrombus formation in cremaster muscle preparations of mice treated with either physiological saline (control), antithrombin (250 IU/kg; AT), tryptophan^49^-blocked AT (250 IU/kg; TrypAT), indomethacin (5 mg/kg; indo), indomethacin plus AT (indo + AT), heparin (100 IU/kg), or heparin plus AT (hep + AT). Data is given as the following single values: Kaplan-Meier-Analysis (Gehan-Breslow Test), * *p* < 0.05 vs. AT

The patency rate is defined as the percentage of patent vessels at a certain time point during TI. The application of physiologic saline and dual TI led to a patency rate of 13 % of investigated vessels after 1200 s (Fig. 3). The application of AT, however, was far more effective as all vessels under investigation were found patent at 600 s (100 %) of continuous TI. After 1200 s of TI, only 62 % of the vessels were clogged resulting in a patency rate of 38 % in the AT group (Fig. 3). TrypAT and indomethacin pre-treatment led to a patency rate of 33 % after 1200 s. The application of heparin and heparin in combination with AT or indomethacin and indomethacin plus AT led to patency rates of about 20 % after 1200 s of continuous TI (Fig. 3).

### Complete vessel occlusion (CVO)

**Fig. 4:**
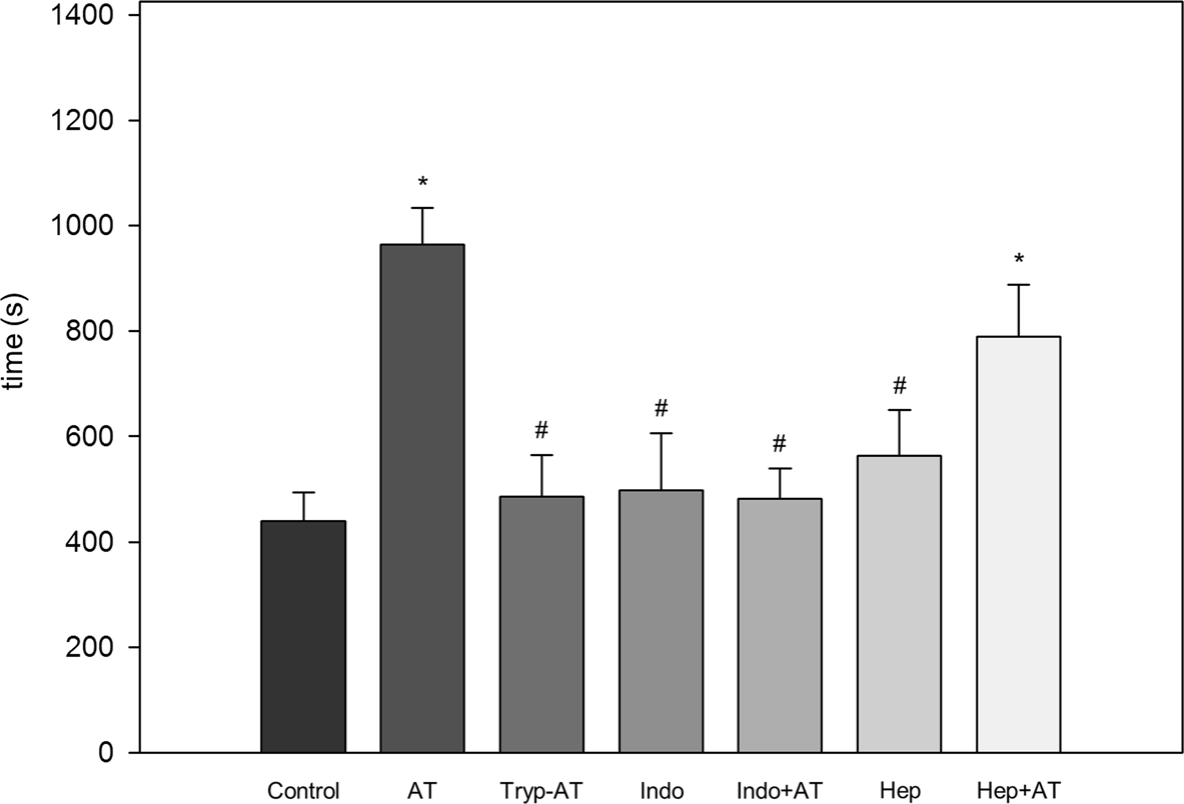
Complete vessel occlusion time. Time until complete occlusion of venules after induction of thrombus formation in cremaster muscle preparations of mice treated with either physiological saline (control), antithrombin (250 IU/kg; AT), tryptophan^49^-blocked AT (250 IU/kg; TrypAT), indomethacin (5 mg/kg; indo), indomethacin plus AT (indo + AT), heparin (100 IU/kg), or heparin plus AT (hep + AT). Data is given as means ± SEM; ANOVA, post-hoc comparison; * *p* < 0.01 vs. control; ^#^ *p* < 0.01 vs. AT

CVO includes all clogged vessels within the investigation time of 1200 s and describes the mean time that was necessary for irreversible occlusion of a vessel by a thrombus (Fig. 4). In control animals, CVO was observed after 440 ± 54 s of ferric chloride and light exposure. AT application presented with major antithrombotic effectiveness, as given by a significant and more than 2-fold prolongation of the time (964 ± 69 s), which was needed for CVO (Fig. 4; *p* < 0.05 vs. control). To further analyze whether the antithrombotic effect is mandatorily linked to the binding of AT to GAGs, TrypAT has been used. Application of TrypAT showed significantly shorter occlusion times than AT (485 ± 79 s), which were comparable to that in controls, indicating that the GAG binding is being considered responsible for the observed anti-inflammatory capability of AT but is also necessary for its anticoagulant function. Additionally, we could observe that the combined application of indomethacin and AT (482 ± 57 s) as well as the indomethacin application alone (497 ± 108 s) could not prolong microvascular thrombus formation as given by similar values for CVO to those in controls. The combination of heparin and AT led to significantly prolonged CVO compared to the control group (790 ± 98 s, *p* < 0.05 vs. control); however, the effect is below the single AT application results.

## Discussion

Microvascular thrombosis is a pathophysiologic key event during sepsis, due to the cross-activation of the coagulation cascade and the systemic pro-inflammatory response. These effects are mainly based on the Virchow’s triad, composed of endothelial injury as well as abnormal blood flow and blood constituents. As the ICU patient in general is at a high risk of thrombosis and benefits from thrombosis prophylaxis, it is still of on-going interest to study thrombus formation under experimental conditions providing new mechanistic insights of the interactions between the coagulation and inflammatory system, giving advice for the optimization of the clinical therapy.

### Methodological considerations

To test the effects of different substances on their potential to prevent thrombus formation, many distinct models exist [13]. The herein used mouse cremaster muscle preparation is a well-characterized and standardized model to study the kinetics of thrombus formation in the microcirculation. It allows the direct visualization of the process of thrombus formation by intravital fluorescence microscopy in both trans- and epi-illumination techniques. Thrombus formation was initiated by two different models. Ferric chloride (FeCl_3_) superfusion is not primarily based on oxidant stress-induced endothelial cell damage. The diffusion of FeCl_3_ through the vessel wall results in denudation of the endothelial cell and in the appearance of ferric-ion filled spherical bodies, which induce platelet adhesion and thrombin formation [14, 15]. Additionally, the light-dye model was used to be able to directly observe the growing thrombus with the intravital microscope [16, 17]. Both models result in local endothelial cell damage leading to the exposure of subendothelial matrix components (tissue factor; P-selectin). This supports the deposition of blood cellular components, representing the ideal site for thrombus formation in micro- as well as macro-vessels [13]. Furthermore, it involves the release/formation of fibrinogen/fibrin, von Willebrand factor, fibronectin, and the glycoprotein IIb/IIIa [18–20]. Therefore, the combination of the light-dye model with the exposure of vessels to ferric chloride for thrombosis induction represents a common and widely used model to study, in particular, molecular and cellular mechanisms of microvascular thrombus formation [21–23] and to study the association between inflammation and thrombosis.

Clinical studies suggest an AT supplementation to achieve a plasma AT activity of >120 % during the observational period [24, 25]. Early studies by Fourrier et al. [26] in septic shock patients with disseminated intravascular coagulation (DIC) received 90–120 IU/kg/d AT. This dosage could already reduce mortality by 44 % in AT-treated patients vs. the control group. The importance of an effective AT plasma activity could additionally be shown in many clinical studies of low AT plasma activity and bad outcomes of septic patients [26–28]. Furthermore, Ranucci et al. [29] could demonstrate that low AT levels in cardiac surgery patients at hospitalization are associated with a significant prolongation of intensive care treatment. Therefore, we have chosen a dose of 250 IU/kg bw which results in plasma concentrations comparable to those achieved in clinical practice and that have been shown to possess therapeutic, antithrombotic, and anti-inflammatory potential in previous studies of our group [10, 11, 30–32]. With this dosing regimen, we have been able to achieve high physiological plasma levels of approximately 120 % after application of 250 IU/kg bw. [11]. In addition, Uchiba and colleagues could demonstrate that lower doses of AT (50 and 100 IU/kg bw) significantly inhibited coagulation abnormalities, but however, failed to prevent pulmonary accumulation of leukocytes and subsequent pulmonary vascular injury [33].

The pre-treatment with indomethacin results in an inhibition of the cyclooxygenase pathway preventing the formation of prostacyclin [34]. In a previous study, we could show that the combination of AT with indomethacin completely abolished the anti-inflammatory effects of AT on the microcirculation [30]. This has been in line with other findings, where AT has been shown to promote prostacyclin release from endothelial cells [35]. Therefore, the primary target cell of the indomethacin pre-treatment was the endothelial cell. However, COX inhibition also targets platelets, which is associated with some prothrombotic tendency [36–38].

### Anticoagulant profile of AT

In patients with trauma, shock, and sepsis, the AT activity is decreased due to its consumption via complex formation with clotting factors and enzymatic degradation [1, 2, 9]. Many experimental as well as clinical trials demonstrated that the supplementation of AT exerts additional anti-inflammatory effects [5, 24, 25, 39–41]; however, in the clinical setting of sepsis therapy, AT is not recommended as high-dose AT was associated with an increased risk of bleeding when administered with heparin [4]. Although heparin is known for its potentiation of the anticoagulant activity of AT [42], it antagonizes simultaneously AT-mediated anti-inflammatory effects [31, 42–44]. This observation was also found during the KyberSept-trial [3, 6, 8] where the combination of AT and heparin failed to improve the mortality of patients with severe sepsis.

While the anti-inflammatory activity of AT seems to be independent from its anticoagulant function [32], our data demonstrates that the effects of AT in the microvascular system crucially depend on its anti-inflammatory mode of action. With the inhibition of the anti-inflammatory activity of AT by a specific blocking of the endproduct synthesis of PGI_2_ (indomethacin) or inhibiting the GAG binding (TrypAT, heparin), the CVO results are comparable with control animals. Binding of AT to endothelial GAGs is consecutively associated with PGI_2_ release. This step has been confirmed by studies, demonstrating the abrogation of the beneficial effects of AT by inhibition of PGI_2_ release with indomethacin in pulmonary vascular injury and systemic endotoxemia [30, 34]. As TrypAT does not display any anti-inflammatory properties due to an ineffective heparin-binding ability at the endothelial surface [45], its administration might enable the discrimination between endothelium-related effects of AT and GAG-independent mediation of anticoagulation [32]. This specific effect of TrypAT has been shown in several previous studies, such as gastric mucosal injury [46], renal injury [47], pulmonary vascular injury [48, 49], and spinal cord injury [50]. Consequently, we used TrypAT to discriminate between the two effects of AT, i.e., anticoagulation and anti-inflammation. In our study, the previous suggestion that native AT and TrypAT might have similar anticoagulant activities, as given by the similar improvement of coagulation abnormalities, could not be confirmed by our in vivo results, showing that TrypAT-treated animals presented significantly faster thrombosis formation compared to animals treated with native AT. This implies the decisive role of the AT-GAG interaction at the microvascular endothelial surface also for the ATs anticoagulant effect. Despite the optimized modification by Blackburn et al. [12] resulting in TrypAT, which is used to differentiate between specific AT effects at the endothelium and in the microcirculation, it cannot be ruled out that also—at least in part—other tryptophane residues might be blocked. This might lead to an alteration of the heparin-dependent acceleration of thrombin-modified AT interactions as well; however, it is not completely abolished [51]. This underscores the results of our study, as we could not confirm the previous suggestion that native AT and TrypAT might have similar anticoagulant activities as given by the similar improvement of coagulation abnormalities [32]. TrypAT-treated animals presented significantly faster thrombosis formation compared to animals treated with native AT, implying the decisive role of the AT-GAG interaction at the microvascular endothelial surface for anticoagulation.

**Fig. 5:**
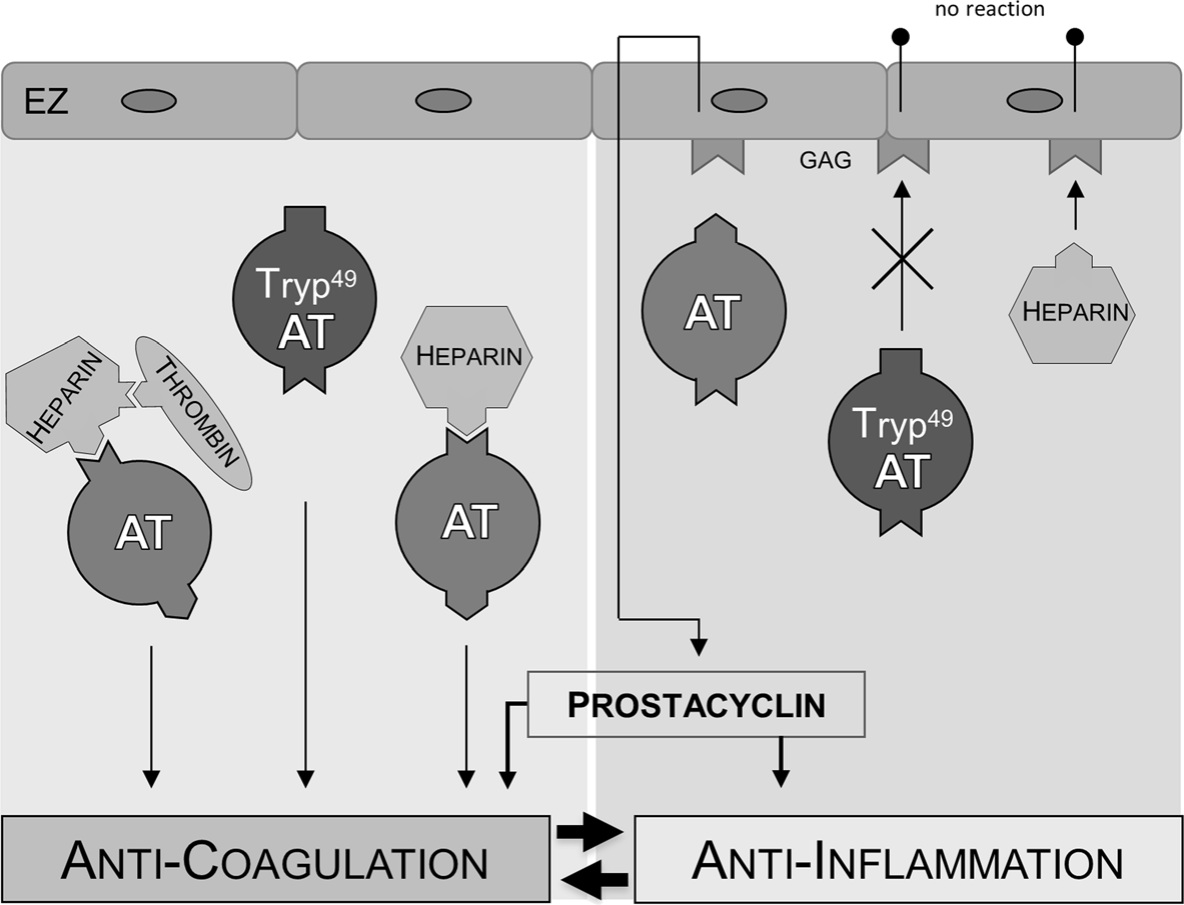
Function of antithrombin. Schematic overview of the anticoagulant function of antithrombin (AT) and the mediation of anti-inflammation through glycosaminoglycan (GAG) binding at the endothelial cell (EC) with subsequent release of prostacyclin (PGI_2_). Until now, it was not clarified whether the antithrombotic potency of AT is exclusively based on its solitary anticoagulant capacity or if it might also depend on its anti-inflammatory mode of action. The data in the here-presented study indicates that the anti-inflammatory property of AT is a prerequisite for mediating adequate anticoagulation

It is well known that AT is a pleiotropic inhibitor of the activated coagulation cascade and that binding of heparin to AT seems to be a prerequisite for enhanced anticoagulant effects [42]. Factor Xa-inhibition by AT deserves a unique heparin pentasaccharide sequence, whereas longer polysaccharide chain heparins are required to enhance the inhibition of thrombin by AT [43, 52, 53]. The observation that the combination of AT plus heparin in our study did not present a prolongation of thrombus formation challenges this view and might be explained as follows. On the endothelial surface, localized GAGs are also comprised of heparan sulfates and heparin-like structures, which exert the same effect on AT as heparin in the blood [42, 54]. These heparin-like binding sites are tenfold better developed in capillaries than in macro-vessels [55, 56], which might be the cause for the microcirculation being the preferential site of AT action. The counteraction of AT by heparin has so far only been described for the anti-inflammatory action, as heparin seems to diminish the vascular defense shield by keeping AT away from its cellular binding sites [42, 43]. While heparin and AT compete for these binding sites, exogenous administration of heparin might interfere not only with the anti-inflammatory but also in part with the anticoagulant activity of AT, as combined administration of heparin and AT was not able to achieve similar protection of thrombus formation as AT alone, which is displayed by the shorter CVO times. Furthermore, the heparin data is in line with previously published results by our own and other groups, showing that heparin in the applied dosage is not associated with changes in venular thrombus formation times [11, 55, 57–59]. However, at higher doses, heparin may also promote the activation of platelets and clot formation by interacting with platelet factor 4 [60, 61]. Our data reveals the essential importance of endothelial AT-GAG interactions for microvascular thrombosis. The competing situation between heparin and AT for endothelial GAGs is of crucial relevance, as AT is not able to trigger the release of PGI_2_ from the endothelium after the GAGs have been irreversibly blocked by heparin (Fig. **5**).

In the context of inflammation and coagulation, there is mounting evidence that both cascades are not independently acting pathways [62–66]. Rather there is a multifaceted crosslink between the coagulation cascade, the inflammatory system, and the local endothelium. The anti-inflammatory potential of AT is directly linked to the cyclooxygenase pathway [32] as in a second hit model of severe endotoxemia the pre-treatment with indomethacin completely abolished the protective effects of AT on leukocyte-endothelial cell interaction and microvascular perfusion failure [30]. Following the results of the here-presented study, the cyclooxygenase pathway which is initiated by the binding of AT to the endothelium, might also be suggested to be essential for the anticoagulant capability of AT as well because indomethacin pre-treatment attenuated AT effects to prolong thrombus formation.

## Conclusions

In conclusion, we have been able to characterize the distinct anticoagulant profile of AT in a murine model of microvascular thrombosis. Our data indicates that the anti-inflammatory potential of AT is indispensable for the adequate mediation of anticoagulation within the microvasculature. These intricate interactions are of important relevance for the therapeutical options of septic disorders and once again support the intimate relationship between the coagulation cascade and the inflammatory system.
